# A Bibliometric Analysis of Low-Cost Piezoelectric Micro-Energy Harvesting Systems from Ambient Energy Sources: Current Trends, Issues and Suggestions

**DOI:** 10.3390/mi13060975

**Published:** 2022-06-20

**Authors:** Mahidur R. Sarker, Mohamad Hanif Md Saad, Amna Riaz, M. S. Hossain Lipu, José Luis Olazagoitia, Haslina Arshad

**Affiliations:** 1 Institute of IR 4.0, Universiti Kebangsaan Malaysia, Bangi 43600, Selangor, Malaysia; hanifsaad@ukm.edu.my (M.H.M.S.); haslinarshad@ukm.edu.my (H.A.); 2 Industrial Engineering and Automotive, Nebrija University, Campus de la Dehesa de la Villa, Calle Pirineos, 55, 28040 Madrid, Spain; jolazago@nebrija.es; 3 Department of Electrical Engineering, Bahauddin Zakariya University, Multan 60000, Pakistan; amna.riaz@bzu.edu.pk; 4 Department of Electrical and Electronic Engineering, Green University of Bangladesh, Dhaka 1207, Bangladesh; shahadat@eee.green.edu.bd

**Keywords:** piezoelectric, energy harvesting, low-cost sensors, low power, low-cost applications

## Abstract

The scientific interest in piezoelectric micro-energy harvesting (PMEH) has been fast-growing, demonstrating that the field has made a major improvement in the long-term evolution of alternative energy sources. Although various research works have been performed and published over the years, only a few attempts have been made to examine the research’s influence in this field. Therefore, this paper presents a bibliometric study into low-cost PMEH from ambient energy sources within the years 2010–2021, outlining current research trends, analytical assessment, novel insights, impacts, challenges and recommendations. The major goal of this paper is to provide a bibliometric evaluation that is based on the top-cited 100 articles employing the Scopus databases, information and refined keyword searches. This study analyses various key aspects, including PMEH emerging applications, authors’ contributions, collaboration, research classification, keywords analysis, country’s networks and state-of-the-art research areas. Moreover, several issues and concerns regarding PMEH are identified to determine the existing constraints and research gaps, such as technical, modeling, economics, power quality and environment. The paper also provides guidelines and suggestions for the development and enhancement of future PMEH towards improving energy efficiency, topologies, design, operational performance and capabilities. The in-depth information, critical discussion and analysis of this bibliometric study are expected to contribute to the advancement of the sustainable pathway for PMEH research.

## 1. Introduction

The research interest in low-cost piezoelectric micro-energy harvesting (PMEH) devices in energy harvesters has grown significantly over the last decade, and it has become one of the most interesting trends in future low-power sensor applications [1,2,3]. The topic of low-cost PMEH has risen to prominence as a fascinating new topic in terms of application and the need for alternative energy sources for the electrical powering of various microelectronics devices [4,5]. This trend has resulted in a massive increase in the number of publications that are extremely important for the future development of the devices. Since several years ago, many research activities have been undertaken and presented in order to highlight the progress of low-cost PMEH development and enhancement, particularly for wireless sensor networks (WSNs), internet of things (IoT), and consumer electronics applications [6,7].

Previous research work on low-cost PMEH using electromagnetic, electrostatic, and multi-frequency mechanical vibration-based electromagnetic energy conversion was published in different publications [1,8,9]. Meanwhile, research on functional materials, such as polymer composites that are applied to an electromagnetic actuator, and the integration of PMEH into the IoT, have significantly expanded the literature in the field of low-cost energy harvester (EH) systems [10,11]. However, just a few attempts have been made to assess the research’s influence in this area. It has also become difficult to understand the research progress on the low-cost PMEH topic, since the number of papers has expanded dramatically over the last decade, despite the lack of a clear direction. As a result, it is critical to conduct a thorough review of PMEH research progress through an analysis of the literature, in order to acquire appropriate directions for future development and research direction. The examination of a publication that highlights current research and development in a topic of concern and provides some guidance for future study is one of the most effective approaches for understanding the research roadmap. It can also help other scholars to avoid duplication of effort or research.

In the analytic revolution in a certain research field, the bibliometric analysis is a powerful and effective approach, rather than a critical review on PMEH giving a clear overview of the global developments [12,13]. Bibliometrics is a research method for analyzing a material quantitatively in a research subject. The analytical evaluation of scientific research publications data using quantitative and qualitative statistical approaches is referred to as analysis. The bibliographic mapping, publication profiling, grouping, and visualization of published works are all part of the investigation [14,15,16]. Furthermore, this study provides a helpful guide for experts to investigate prevalent interrelationships in the specific literature, as well as the impact of title, journals, researchers, countries, and organizations. The study may also be able to assist researchers in predicting future research trends.

Bibliometrics investigates numerous aspects of texts, such as co-relationship, paper structure, and paper strategy, using statistical and mathematical methodologies. A bibliometric analysis can also be used to assess different features of a certain study topic. Bibliometrics were applied to examine climate change vulnerability, haze study, COVID-19 research, and low carbon education research [17,18,19]. This study is a comprehensive analysis that was carried out in the field of PMEH in the last decade with complete information and the effect of all dependent parameters. Consequently, it is difficult to find PMEH research by identifying and summarizing it from all the manuscripts that were published in the last decade. This manuscript helps to evaluate the impact of different publications in the field of PMEH. Therefore, based on this analysis, the future direction of research and trends in the field of PMEH is clear. Table 1 presents a summarized analytical evaluation of the papers that were generated by numerous scholars in the field of PMEH, as well as research gaps [20,21].

The most important key findings and conclusions of this study are summarized below:

- Mathematical modelling, algorithm creation, data collecting, and simulation for EH are the most common categories of study in published manuscripts (35.38 percent);
- The most cited authors, the universities with the most publications, and the country with the most published articles on the mentioned topic are used to examine power converters for PMEH applications. This is critical for determining author, organization, and country productivity in the discussed topic, as well as improving research output and collaboration among authors;
- The content and gap analysis keywords and themes are evaluated;
- The analyzed documents are original manuscripts, review articles, and book chapters. The journals’ impact factors and the repute of the publishers in the scientific community are also investigated.
- The extent of researcher collaboration is determined. The team is evaluated based on the number of authors in the manuscripts and the connections between different universities and countries. The goal of the developing trends and analytical assessment is to look at the top 100 papers in low-cost PMEH. As a result, thorough and detailed information, critical arguments, facts, analyses and flaws, issues, and challenges relating to these publications are discussed. This review has several advantages, including:
- To present PMEH in low-cost applications, as well as current trends, evolution, applications, and future research potential;
- To give a comparative assessment of the most appropriate publications for PMEH in low-cost applications that will help to expand current knowledge, experience, and implementation in the future;
- Finally, this analytical study offers helpful recommendations for PMEH’s future development and possibilities in low-cost applications.

The remainder of the paper is organized as follows: Section 2 discusses the methodological screening procedure of this study and how Table 2 was extracted from the Scopus database, as well as the publishing trend in this subject and the research characteristics. Section 3 discusses the analytical discussion related to the most-cited papers, the co-occurrence of keywords, most prominent authors’ affiliation, most prominent journal, study features, and other subject areas. Section 4 presents the issues and challenges of a low-cost PMEH system and the final section contains closing remarks and recommendations for future studies.

## 2. Review Methodology

The research survey was conducted in the Scopus database during the third week of December 2021, looking for articles that were published in journals indexed between 2010 and 2021, in order to gain complete information about the latest scientific research in the area of “low cost PMEH” from abundant energy resources. The primary goal of this study is to portray the latest picture of technological advancement and revolution in PMEH as it relates to power sources that generate power with low cost from abundant energy resources in the recent publications [26]. To choose the highly cited papers, we needed to search various criteria. In this manuscript, Bibliometric, which is a statistical study of the Scopus database, was employed for evaluation (www.scopus.com, 30 December 2021). Since it includes a larger number of publications than other databases, such as Web of Science, the Scopus database is the source of analysis in this analytical evaluation survey [27,28]. Google Scholar does not return precise results; for this reason, we do not consider Google Scholar in this survey. The “low cost PMEH” study was monitored in the Scopus database at the end of December 2021. 

### 2.1. Literature Screening

A comprehensive report that is published in Scopus highlights the research output in PMEH from 2010 to 2021. There are three steps in the complete paper-screening procedure. To begin, all Scopus articles about PMEH are gathered as the primary input, which is linked with the title, abstract, and keywords of any phrases that are based on PMEH (such as microelectronic devices, abundant energy resources, and clean energy) [29,30]. The publication took place between 2010 and 2021.

**Figure 1:**
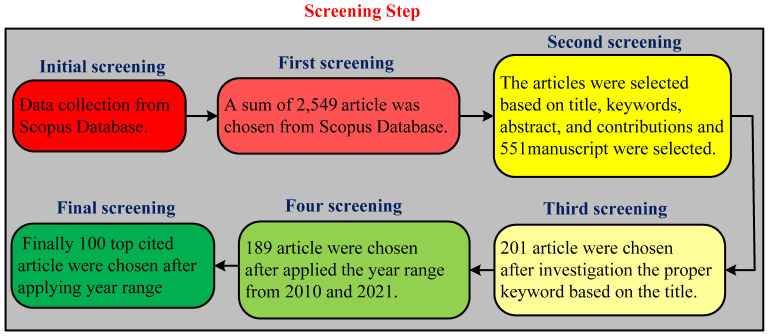
Block diagram of the reviewing methodology for overall article selection process.

Secondly, 551 papers covering numerous subjects, such as renewable energy resources, power harvesting, piezoelectric EH, and energy were initially identified. Finally, the first-recognized papers are divided into numerous categories, including journal articles, conference papers, books, reviews, and other sorts of publications for debate and additional study. Figure 1 depicts the overall selecting-procedure analytical evaluation approaches that were applied in the Scopus database. As illustrated in the image below, the process is divided into five steps:

### 2.2. Selection and Exclusion Criteria

The articles were chosen using predetermined criteria from a Scopus database. The initial searches for keywords that were utilized for the related manuscript in the Scopus database are listed in Table 2. The top 100 most-cited manuscripts in the subjects of low-cost EH, piezoelectric EH, low-cost electronic devices, low-cost applications that were chosen for article inclusion and exclusion were used:

- The following keywords were used as the major criteria for including manuscripts: low-cost PMEH, low-cost electronics, low-cost applications, low-cost sensors, and low-cost controllers. Some articles were left from the list due to the field’s insignificance.
- Manuscripts that were published in the English language from the years 2010 to 2021 were considered for the study’s aims.

### 2.3. Screening Procedures

Due to the differences in output for each database, compiling a single list of each article was impossible. The Scopus database was used for the screening process. For each keyword, the five screening steps were used, as shown below.

A varying number of articles were found in the Scopus database following the main search for “PMEH, renewable energy resources, and low cost control systems,” and it was comprehensive at different time frames.

- The first screening was limited to the “EH system for low power applications” and 2549 articles were selected.
- The articles were selected based on titles, keywords, the abstract, and contributions, and 551 manuscripts were selected.
- The 201 articles were chosen after investigating the proper title and abstract.
- The fourth screening selected 189 articles between the years 2010 and 2021.
- Finally, the 100 top-cited articles are selected based on the year range.

### 2.4. Research Trend

**Figure 2:**
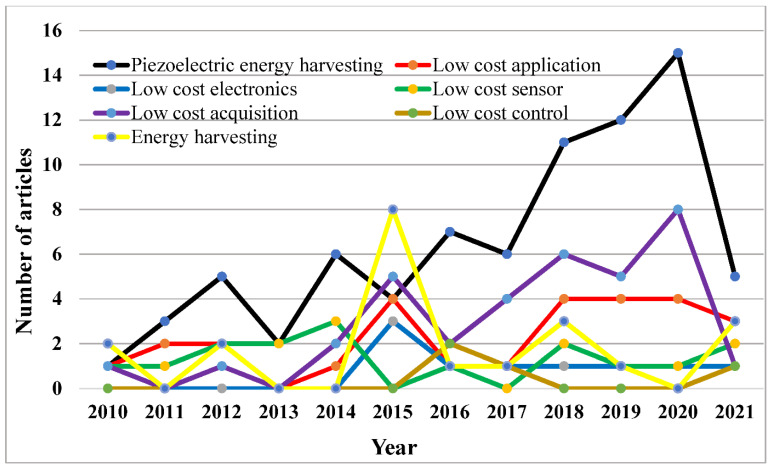
The frequency of manuscripts per year.

The Scopus database was searched for all published papers using the following search terms: “renewable energy resources, low cost PMEH, low cost electronics, low cost application, and low cost control”. Figure 2 shows the research publications in the area of PMEH from the year 2010 to 2021.

Figure 2 shows that the search phrase “piezoelectric energy harvesting” yields the most publications, followed by EH, low-cost acquisition, low-cost applications, low-cost sensors, and low-cost control. The results of various papers differ from year to year. Overall, the patterns suggest an increase in the frequency of published manuscripts from 2010 to 2021, followed by a reduction in 2021.

The expanding number of articles on “piezoelectric EH” suggests that it is now widely used and accepted in engineering research. In the future, the industry’s growth tendency is likely to be a major development. However, since only the data for January 2021 were collected, it is too early to anticipate for 2021. The articles on piezoelectric EH, low-cost application, and low-cost acquisition are all at the top of the research papers that were released in 2020. The largest number of articles that were published in 2020 for piezoelectric EH was 15. However, the published papers for piezoelectric EH show the maximum number of 17 publications between 2017 and 2018. It is also an important point that papers were published on piezoelectric EH throughout 2010 to 2021.

### 2.5. Study Characteristics and Outcomes

This study looks at alternative search phrases in the Scopus database for the same-discipline analysis. From an analytical evaluation that is based on six keywords, we want to search manuscripts that are related to “low cost EH”. From 2010 to 2021, a total of 2549 papers were found in the Scopus database. All of the authors come from different backgrounds and nations, yet they all work on similar engineering problems. The low-cost PMEH and other databases are discussed in several bibliometric works in the literature. Empirical research with these findings can help in the future development of PMEH. The number of citations, keywords, the topic, publishing year, publisher, document type, impact factor, author’s h-index, university’s h-index, and authors with the more cited publications, organizations, and nations are all considered in this section. Finally, the bibliometric analysis is completed.

### 2.6. The Trends of Publishing Manuscripts per Year

**Figure 3:**
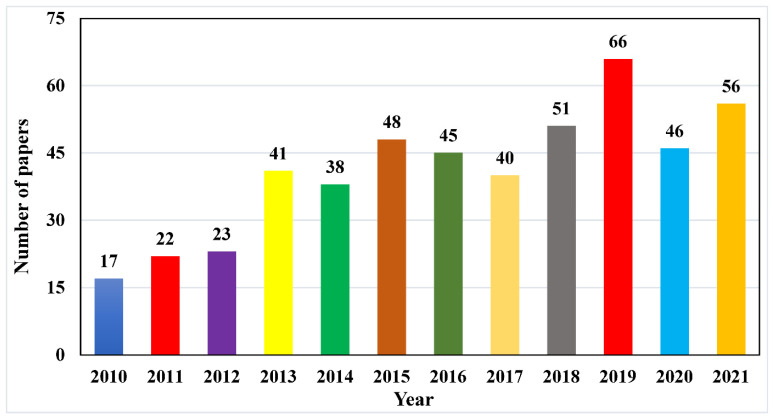
Before scanning the frequency of published manuscripts per year.

Figure 3 depicts the frequency of manuscripts that were published from 2010 to 2021. Before scanning methods, a total of 493 (Figure 3) papers were examined. As can be seen, the number of papers that are released gradually increases until 2019, when there are approximately 66 papers. The rise in research demonstrates the industry’s acceptance and significant potential for development. Nonetheless, the quantity of publications has decreased slightly in recent years and will continue to do so through 2020. The number of published papers has reduced in previous years as the article number for January 2021 is yet to be determined. These data appear to be dynamic, and if maturity is achieved, the tendencies will shift in the future.

The most significant issue is to evaluate the publications with most referenced papers. The evaluation data for 100 publications on EH are presented in Table 3. The author’s name, article DOI, keywords, types of articles, abbreviated name, publisher, publishing year, country of origin, number of citations, and impact factor are used to classify the data. As can be observed, Chang et al. [31] have the most frequent citations (992). The title of the article refers to the “Direct-write piezoelectric polymeric nanogenerator with high energy conversion efficiency”. The purpose of this paper is to highlight the conversion of mechanical energy into electrical energy with the properties of high efficiency and low cost when nano semiconductors are used in EH. The paper was published in the American Chemical Society as a journal article. With 844 citations, a study by Mao et al. [32] is reported to be the second-highest citation article. With the keywords EH, low-cost electronics, and low-power, the topic of the paper is the investigation of green mobile edge-computing with an EH system [33]. The study in [34] reported a third high-citation publication in 2010. The title of the study is “Structural health monitoring of a cable stayed bridge using smart sensor technology: deployment and evaluation”. This paper is about the structural monitoring of the civil infrastructures with the help of WSNs. This system is low cost, has installation simplicity, and provides effective data management.

The top papers include Dong et al. [35], Fan [36], and Hu et al. [37], all of which have over two hundred citations. The impact factors of these three journals are 9.127, 15.881, and 17.881, respectively. Among 100 papers, 14 percent have received more than 100 citations, 14 percent have received more than 50 citations, while the remaining 72 percent have 50 citations or less. Nevertheless, the frequency of citations for a specific study is not the best way to indicate the quality of the research work [38,39,40]; however, it expresses the readership of the individual work and its impact on the creation of interventions, controversy, discussion, and some additional studies. This is also an advantage of the paper in the field.

Citation analysis has become a well-known method of determining manuscripts’ or authors’ impact on a certain topic in a publication [41,42,43]. The goal of this work was to determine the characteristics of low-power PMEH research. We have included the paper in the research even though it has a minimal number of citations. The top 100 cited articles were reviewed and debated, and it was discovered that the bulk of recent studies focused on the low-cost EH, low-cost electronics, and applications. The overall survey indicated that low-cost PMEH is important, not only as a power source, but also for health and structural monitoring. In terms of market potential, piezoelectric is advantageous for EH.

**Table 3:** The list of 100 highly influential papers for energy harvesting keywords and publication detail.

Rank Based on Citation	References	Author Name	Article DOI	Keywords	Type of Article	Abbreviated Name	Publisher	Publishing Year	Country	NC	IF
1	[31]	Chang	10.1021/nl9040719	EH, PZT, low-cost acquisition	Article	Nano Letter	American Chemical Society	2010	USA	992	11.189
2	[32]	Mao	10.1109/JSAC.2016.2611964	EH, low-cost electronics, low power	Article	ISACE	IEEE	2016	USA	844	9.144
3	[34]	Jang	10.12989/sss.2010.6.5_6.439	EH, low-cost sensors, WSN, low power	Article	Smart. Struct. Syst.	Techno Press	2010	South Korea	355	3.342
4	[35]	Dong	10.1016/j.jpowsour.2011.01.090	EH, PZT, low-cost applications	Review	J. Power Sources	Elsevier	2011	The Netherlands	352	9.127
5	[36]	Fan	10.1021/acsnano.5b00618	EH, self-power, WSN, low-cost electronics	Article	ACS Nano	American Chemical Society	2015	USA	266	15.881
6	[37]	Hu	10.1016/j.nanoen.2014.11.038	EH, PZT, low-cost sensors, power	Article	j.nanoen	Elsevier	2014	The Netherlands	237	17.881
7	[44]	Martinez	10.1109/JSEN.2015.2445094	EH, Low-cost electronics, low power, WSN	Article	IEEE Sens. J.	IEEE	2015	USA	173	3.301
8	[45]	Lee	10.1002/adfm.201202867	EH, PZT, low-cost sensors, self-power	Article	Adv. Funct. Mater.	WILEY	2013	Germany	190	18.808
9	[46]	Garain	10.1021/acsami.5b11356	Low power, EH, PZT, sensor	Article	ACS AMI	American Chemical Society	2016	USA	147	9.229
10	[47]	Thielen	10.1016/j.enconman.2016.11.005	EH, low power, low-cost electronics, optimization	Article	ECMAD	Elsevier	2017	England	123	9.709
11	[48]	Wang	10.1109/TMC.2017.2732979	EH, low-cost sensors, low power, WSN	Article	IEEE TMC	IEEE	2018	USA	110	5.538
12	[49]	Ghosh	10.1016/j.nanoen.2017.04.028	PZT, sensors, self-power	Article	Nano Energy	Elsevier	2017	USA	109	17.881
13	[50]	Qiu	10.1039/c2nr31031g	Low-cost application, PZT, low power	Article	Nanoscale	Royal Society of Chemistry	2012	England	102	7.790
14	[51]	Liang	10.1080/15583724.2010.515765	EH, low-cost application, low-power devices	Article	Polym. Rev	Taylor and Francis	2010	USA	101	13.282
15	[52]	Kornbluh	10.1557/mrs.2012.41	EH, low-cost acquisition, electronics devices	Article	MRSBE	Springer	2012	Germany	95	6.578
16	[53]	You	10.1039/c7ta10175a	PZT, low-cost acquisition, self-powered	Article	JMCAE	Royal Society of Chemistry	2018	England	85	12.732
17	[54]	Zhang	10.1016/j.nanoen.2017.01.053	Low-cost control, low power, autonomous sensors	Article	Nano Energy	Elsevier	2017	The Netherlands	85	17.881
18	[55]	Dudem	10.1016/j.apenergy.2018.09.009	PZT, EH, low-cost acquisition	Article	APEND	Elsevier	2018	England	78	9.746
19	[56]	Lee	10.1088/0964-1726/23/9/095044	Low-cost sensor, PZT, EH	Article	SMSTE	Institute of Physics Publishing	2014	England	72	3.585
20	[57]	Todaro	10.1016/j.mee.2017.10.005	PZT, EH, low power, low-cost acquisition	Review	MIENE	Elsevier	2017	The Netherlands	72	2.523
21	[58]	Nunes-Pereira	10.1016/j.compositesb.2014.12.001	EH, low-cost acquisition, power, sensor	Article	CPBEF	Elsevier	2015	England	67	9.078
22	[59]	Park	10.1186/s40580-016-0072-z	PZT, low-cost sensor, EH, low power	Review	Nano Converg.	Korea Nano Technology Research Society	2016	USA	61	8.526
23	[60]	Lu	10.1016/j.nanoen.2020.105251	PZT, low-cost acquisition, EH	Review	Nano Energy	Elsevier	2020	The Netherlands	60	17.881
24	[61]	Han	10.1109/TIE.2014.2383992	EH, low-cost applications, low power	Article	ITIED	IEEE	2015	USA	60	8.236
25	[62]	Datta	10.1002/adfm.201604262	PZT, low-cost acquisition, EH	Article	AFMDC	Wiley	2017	Germany	59	18.808
26	[63]	Lazaro	10.3390/s18113746	EH, low-cost electronics, IoT devices, low power	Review	Sensors	MDPI	2018	Switzerland	56	3.576
27	[64]	De Pasquale	10.1115/1.4006920	EH, vibration, low power, low-cost control	Article	JCND	ASME	2012	USA	54	2.085
28	[65]	Sun	10.1016/j.nanoen.2018.03.071	PZT, low-cost sensor, EH, low-power electronic	Article	Nano Energy	Elsevier	2018	The Netherlands	53	17.881
29	[66]	Awais	10.1109/ACCESS.2018.2848907	EH, WSN, low-cost acquisition	Article	IEEE Access	IEEE	2018	USA	50	3.367
30	[67]	Vertechy	10.1115/1.4028508	EH, low-cost acquisition, low power	Article	JVACE	ASME	2015	USA	46	1.583
31	[68]	Hänninen	10.1016/j.carbpol.2018.09.001	PZT, low-cost acquisition, EH	Article	CAPOD	Elsevier	2018	England	49	9.381
32	[69]	Jing	10.1088/1361-6463/aac827	EH, PZT, low-cost acquisition, electric power	Review	JPAPB	Institute of Physics Publishing	2018	England	46	3.207
33	[70]	Paprotny	10.1109/JSEN.2012.2211868	EH, PZT, low-cost sensor, power electronic	Article	IEEE Sens. J.	IEEE	2013	USA	46	3.301
34	[71]	Jeon	10.1016/j.nanoen.2015.08.002	EH, low-cost acquisition, vibration, low power	Article	Nano Energy	Elsevier	2015	The Netherlands	43	17.881
35	[72]	Sarker	10.1016/j.sna.2019.111634	EH, PZT, low-cost application, optimization, WSN	Review	SAAPE	Elsevier	2019	Switzerland	42	3.407
36	[73]	Prashanthi	10.1109/JMEMS.2011.2178118	Low-cost applications, PZT, Sensor	Article	JMIYE	IEEE	2012	USA	41	2.417
37	[74]	La Rosa	10.3390/s19122660	EH, low-cost application, low power, WSN	Article	Sensors	MDPI	2019	Switzerland	41	3.576
38	[75]	Nour	10.1016/j.nanoen.2014.07.014	PZT, EH, low-cost acquisition	Article	Nano Energy	Elsevier	2014	The Netherlands	40	17.881
39	[76]	Crossley	10.1179/1743284714Y.0000000605	PZT, EH, Low-cost acquisition, low power	Article	MSCTE	Maney	2014	England	37	0.562
40	[77]	Ando	10.1109/JSEN.2014.2386392	Low-cost electronics, EH, vibration	Article	IEEE Sens. J.	IEEE	2015	USA	36	3.301
41	[78]	Tentzeris	10.1109/JPROC.2014.2361599	Low-cost sensor, EH, PZT, low power	Review	IEEPA	IEEE	2014	USA	33	10.961
42	[79]	Cherumannil	10.1016/j.nanoen.2017.08.052	Low-cost acquisition, EH, PZT	Article	Nano Energy	Elsevier	2017	The Netherlands	31	17.881
43	[80]	Liu	10.1002/admt.201900744	Low-cost electronics, EH, PZT	Article	Adv. Mater. Technol.	Wiley	2019	USA	27	7.848
44	[81]	Song	10.1039/d0ta08642h	Low-cost applications, EH, PZT	Review	JMCAE	Royal Society of Chemistry	2021	England	26	12.732
45	[82]	Le	10.1016/j.jallcom.2020.156172	Low-cost applications, EH, PZT, low power	Review	JALCE	Elsevier	2020	Switzerland	24	5.316
46	[83]	Sun	10.1021/acsnano.0c05493	Low-cost applications, EH, PZT, sensors	Article	ACS Nano	American Chemical Society	2020	USA	23	15.881
47	[84]	Han	10.1109/JSEN.2017.2747122	EH, vibration, PZT, low-cost applications	Article	IEEE Sens. J.	IEEE	2017	USA	23	3.301
48	[85]	Bhunia	10.1021/acsami.9b13360	Low-cost electronics, EH, PZT, power	Article	ACS Appl. Mater. Inter	American Chemical Society	2019	USA	20	9.229
49	[86]	Khansur	10.1016/j.ceramint.2018.06.027	Low-cost acquisition, EH, PZT, sensors	Article	CINND	Elsevier	2018	England	20	4.527
50	[87]	Kang	10.1016/j.nanoen.2015.09.004	Low-cost acquisition, EH, PZT, sensors	Article	Nano Energy	Elsevier	2015	The Netherlands	20	17.881
51	[88]	Algieri	10.1021/acsaem.8b00820	Low-cost applications, EH, PZT	Article	ACS AEM	American Chemical Society	2018	USA	19	6.024
52	[89]	Rajagopalan	10.1088/1361-6528/aaa6bd	Low-cost applications, EH, PZT, sensors	Article	NNOTE	IOP	2018	England	19	3.874
53	[90]	Shivashankar	10.1088/1361-665X/ab7541	Low-cost acquisition, EH, PZT	Review	SMSTE	IOP	2020	England	17	3.585
54	[91]	Maria	10.1016/j.compositesb.2018.12.129	Low-cost application, EH, PZT, sensors	Article	CPBEF	Elsevier	2019	England	17	9.078
55	[92]	Liu	10.1016/j.apenergy.2018.09.051	EH, PZT, low power	Article	APEND	Elsevier	2018	England	16	9.746
56	[93]	Prashanthi	10.1002/pssr.201105538	EH, PZT, low-cost sensor	Article	Phys. Status Solidi-Rapid Res. Lett.	Wiley	2012	Germany	16	2.821
57	[94]	Meddad	10.1063/1.4751456	Low-cost sensors, EH, PZT	Article	JAPIA	AMER INST PHYSICS	2012	USA	15	2.546
58	[95]	Charoonsuk	10.1039/c9tc01622h	Low-cost applications, EH, PZT	Article	JMCCC	Royal Society of Chemistry	2019	England	14	7.393
59	[96]	Singh	10.1088/2053-1591/3/7/075702	Low-cost acquisition, EH, PZT	Article	Mater. Res. Express	IOP	2016	England	14	1.620
60	[97]	Hu	10.1177/1045389X13489781	Low-cost applications, EH, PZT	Article	JMSSE	Sage	2014	England	14	2.569
61	[98]	Gong	10.1016/j.energy.2019.115983	Low-cost electronics, EH, PZT, low power	Article	Energy	Elsevier	2019	England	13	7.147
62	[99]	Kar	10.1021/acsanm.8b00770	Low-cost applications, EH, PZT, power	Article	ACS ANM	American Chemical Society	2018	USA	12	5.097
63	[100]	Nour	10.1002/pssa.201600142	Low-cost applications, EH, PZT, sensor, low power	Article	PSSAB	Wiley	2016	Germany	12	1.981
64	[101]	Vázquez	10.3390/ma12223725	Low-cost acquisition, EH, PZT, low power	Article	Materials	MDPI	2019	Switzerland	11	3.623
65	[102]	Yu	10.1002/mame.201700214	Low-cost applications, EH, PZT	Article	MMENF	Wiley	2017	Germany	10	4.367
66	[103]	Marinkovic	10.1063/1.3524271	EH, PZT, low-cost sensor	Article	JAPIA	American Institute of Physics	2011	USA	10	2.546
67	[104]	Zhao	10.1007/s10854-021-06027-w	EH, PZT, sensors, low-cost sensor	Article	JMSME	Springer	2021	The Netherlands	9	2.478
68	[105]	Manikandan	10.1088/1361-6528/ab6b9e	EH, PZT, low-cost acquisition	Article	NNOTE	IOP Publishing	2020	England	9	3.874
69	[106]	Clementi	10.1016/j.ymssp.2020.107171	EH, PZT, low-cost acquisition, vibration	Article	MSSPE	Elsevier	2021	England	8	6.823
70	[107]	Rjafallah	10.1177/0021998318788604	EH, PZT, vibration, low-cost acquisition	Article	JCOMB	Sage	2019	England	8	2.591
71	[108]	Kandpal	10.1109/TNANO.2017.2659383	low-cost acquisition, PZT, EH	Article	IEEE Trans. Nanotechnol	IEEE	2017	USA	8	2.570
72	[109]	Aboubakr	10.1080/15421406.2015.1137148	low-cost acquisition, PZT, EH	Article	MCLCD	Taylor and Francis Inc	2016	England	8	0.896
73	[110]	Lewis	10.1080/00150193.2012.676955	low-cost control, PZT, EH	Article	FEROA	Taylor and Francis	2012	England	8	0.620
74	[111]	Tu	10.1021/acsami.0c16207	low-cost acquisition, PZT, EH applications	Article	ACSAMI	American Chemical Society	2020	USA	7	9.229
75	[112]	Anand	10.1016/j.jallcom.2020.156019	low-cost acquisition, PZT, EH, low power	Article	JALCE	Elsevier	2020	Switzerland	7	5.316
76	[113]	Vivekananthan	10.1016/j.apsusc.2020.145904	PZT, EH, low power	Article	ASUSE	Elsevier	2020	The Netherlands	7	6.707
77	[114]	Chinya	10.1016/j.materresbull.2019.110515	low-cost acquisition, PZT, EH, vibration	Article	MRBUA	Elsevier	2019	England	7	4.641
78	[115]	Amoroso	10.12989/sss.2015.16.3.383	Low-cost application, PZT, EH, WSN	Article	Smart. Struct. Syst.	Techno-Press	2015	South Korea	7	3.342
79	[116]	Sarker	10.3390/mi7100171	PZT, EH, optimization, low-cost control	Article	Micromachines	MDPI	2016	Switzerland	7	2.891
80	[7]	Sarker	10.1080/00150193.2017.1359028	PZT, EH, optimization, low-cost control, low voltage	Article	FEROA	Taylor and Francis	2016	England	7	0.620
81	[117]	Gao	10.1002/advs.202101834	low-cost applications, PZT, EH, low frequency	Article	Adv. Sci.	Wiley	2021	USA	6	16.806
82	[118]	Pei	10.1016/j.jclepro.2020.125338	low-cost sensor, EH, low power	Review	JCROE	Elsevier	2021	England	6	9.297
83	[119]	Tamil	10.1142/S0219581X1950008X	low-cost acquisition, PZT, EH application	Article	Int. J. Nanosci	World Scientific Publishing	2020	Singapore	6	0.68
84	[120]	Poulin	10.1016/j.mssp.2018.12.013	low-cost acquisition, EH, low power, PZT	Review	Mater. Sci. Semicond. Process	Elsevier	2019	England	6	3.927
85	[121]	Yang	10.3390/s18113733	low-cost application, EH, PZT, low-cost devices	Article	Sensors	MDPI	2018	Switzerland	6	3.576
86	[122]	Wang	10.1177/1045389X14549866	low-cost applications, EH, PZT, vibration,	Article	JMSSE	Sage	2015	England	6	2.569
87	[123]	Lei	10.1063/1.4921832	EH, PZT, low-cost acquisition, devices, vibration	Article	JRSE	American Institute of Physics	2015	USA	6	2.219
88	[124]	Chauhan	10.1016/j.sna.2020.111879	EH, PZT, low-cost sensor, WSN	Review	SAAPE	Elsevier	2020	Switzerland	5	3.407
89	[125]	Erturun	10.1063/5.0030302	EH, PZT, low-cost applications, low-power devices	Article	APPLA	American Institute of Physics	2021	USA	4	3.791
90	[3]	Le	10.1016/j.sna.2020.112148	EH, PZT, low-cost applications, low-power applications	Review	SAAPE	Elsevier	2020	Switzerland	4	3.407
91	[126]	Quattrocchi	10.1109/TIM.2020.3026462	EH, PZT, low-cost electronics, low power, vibration	Article	IEIMA	IEEE	2020	USA	4	4.016
92	[127]	Guiffard	10.1007/s00339-014-8600-3	EH, PZT, low-cost applications	Article	APAMF	Springer	2015	Germany	4	2.584
93	[128]	Chuo	10.1109/JSEN.2011.2160337	EH, PZT, low-cost applications, micro-sensors	Article	IEEE Sens. J	IEEE	2011	USA	4	3.301
94	[129]	Xia	10.1088/1361-665X/aba48d	EH, PZT, self-power, sensors, low-cost acquisition	Article	SMSTE	IOP Publishing	2020	England	3	3.585
95	[130]	Tabhane	10.1016/j.polymertesting.2020.106564	low-cost acquisition, EH, PZT, energy storage	Article	POTED	Elsevier	2020	England	3	4.282
96	[131]	Lozoya-Santos	10.3390/app10124387	EH, PZT, low-cost, low-power applications	Article	Appl. Sci	MDPI	2020	Switzerland	3	2.679
97	[132]	Dietze	10.1002/mame.201900538	EH, PZT, low-cost sensor	Article	MMENF	Wiley	2019	Germany	3	4.367
98	[133]	He	10.1007/s11664-019-07025-9	EH, PZT, low-cost acquisition	Article	JECMA	Springer	2019	USA	3	1.938
99	[1]	Sarker	10.3390/electronics10091108	EH, low power, WSN, low-cost control	Review	Electronics	MDPI	2021	Switzerland	3	2.397
100	[4]	Riaz	10.3390/s21155041	Micro EH, low-cost electronics, WSN, energy storage	Review	Sensors	MDPI	2021	Switzerland	3	3.576

NC is the number of citations, IF is the impact factor.

**Figure 4:**
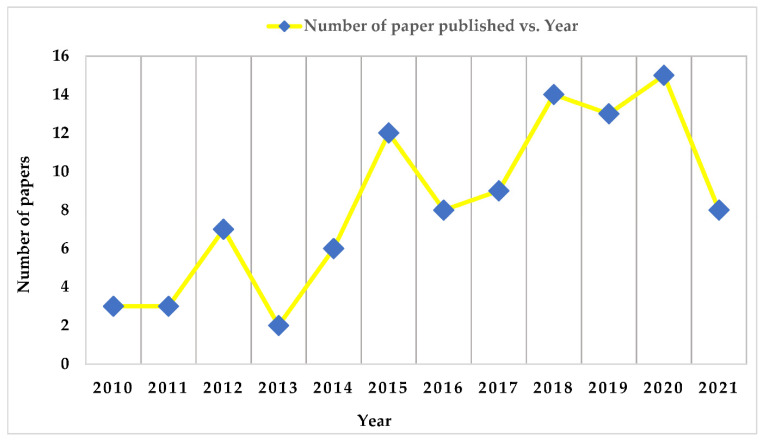
Distribution of 100 top-cited manuscripts from the year 2010 to 2021.

A graphical representation of the total number of publications versus years is pro-vided in Figure 4. The data clearly show that manuscripts that were published in 2016 and 2017 accounted for only 50 percent and 62.5 percent of total publications, respectively. Figure 4 depicts the 16 most-cited manuscripts per year in the area of low-cost PMEH from 2010 to 2021. Figure 4 shows that the year 2013 had the fewest papers published (2), followed by 2010 and 2011 with three articles. As a result, it can be stated that as the age of publications increases, so does the quantity of citations.

## 3. Evaluation and Outcomes

To discover and extend a topic, it is necessary to identify and comprehend recent research trends, as well as the most notable research in that discipline. The goal of this study is to figure out how important current research trends are, as well as to find important articles on the subject of PMEH.

### 3.1. PMEH Application on the Different Research Areas

**Figure 5:**
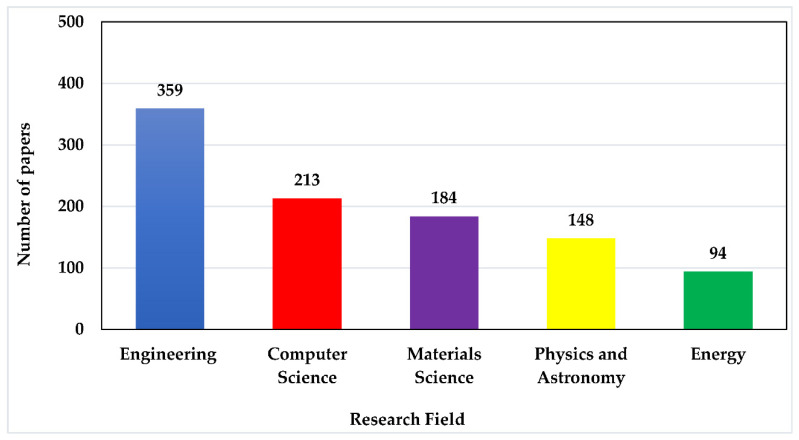
Top 5 research areas in piezoelectric energy harvester for low-power application.

When low-cost PMEH, low-cost electronics, low-cost applications were entered in numerous areas of engineering, computer science, physics and astronomy, material science, and energy, 808 papers surfaced based on the classifications of the gathered articles. The paper classification screening is carried out in this phase using the title, abstract, and keywords. Nonetheless, it is not easy to analyze a work in a specific sector because a manuscript in the PMEH topic incorporates interdisciplinary concerns. The core topic, for example, in engineering is computer science, although physics and materials science are also vital to consider. As a result, the article is divided into four categories: engineering, computer science, material science, and physics. Figure 5 shows the top five fields in PMEH research from 2010 to 2021, according to this guideline. The research was demonstrated to encompass a wide range of topics, including engineering, computer science, material science, physics, and energy. As a result, it is clear that engineering, with 359 papers (44.4 percent of the total screened papers), is the most popular field, followed by computer science, material science, physics and astronomy, and energy.

It is also evident that materials and physics have more papers than energy, indicating that basic science continues to dominate this field of study. Table 4 lists the most referenced publications based on the more prolific authors, country of origin, and number of articles, h-index, and author position. The author with the highest (eight) number of referenced papers is Magno of Switzerland in the university of ETH Zürich. With seven articles, Tentzeris from the Georgia Institute of Technology is the author with the second most citations. Table 4 shows that the USA is ranked second, third, and fourth in this ranking. From the United States, Wang et al. have the top citations (239,581) and the greatest h-index (240). Tentzeris et al. have the second highest number of citations (16,749) and an h-index of 62.

### 3.2. Countries Researching in Low-Cost PMEH

**Figure 6:**
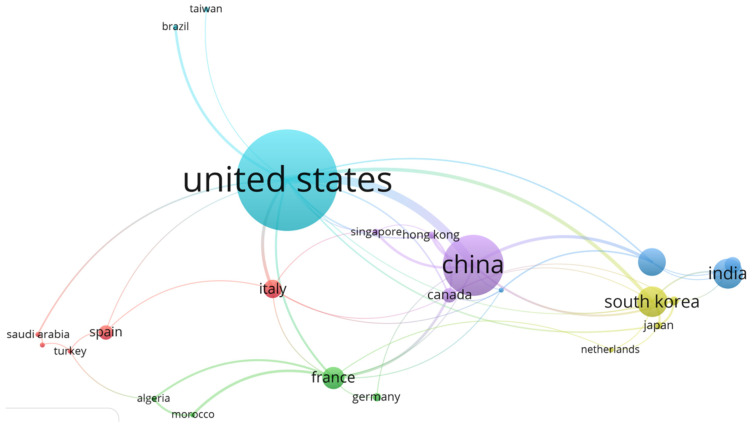
The country’s networks in the research of PMEH with low-cost applications.

The energy supply to operate the low voltage electronic device has become a global problem as the component of micro-electronic products has become smaller and more compact [134,135]. As a result, it is critical to examine articles on EH-device research that have been published in various countries. Figure 6 depicts the distribution of nations where low-cost PMEH research is carried out. Over the last decade, the USA has been the most active in conducting low-cost PMEH research. A total of 31 papers were provided by USA scholars, accounting for 60.8 percent of the total papers that were examined (in Figure 6 this is indicated by a sea-green color). This is acceptable because the USA’s demand for microelectronic devices has increased rapidly over the previous decade and so it is closely tied to low-cost PMEH requirements. China has the second highest publications, indicated by a purple circle; India has publications in the third position with a baby-blue color; and South Korea is in fourth position with an olive-green color in Figure 6.

### 3.3. Journal Publication and Impact Factor Evaluation

**Figure 7:**
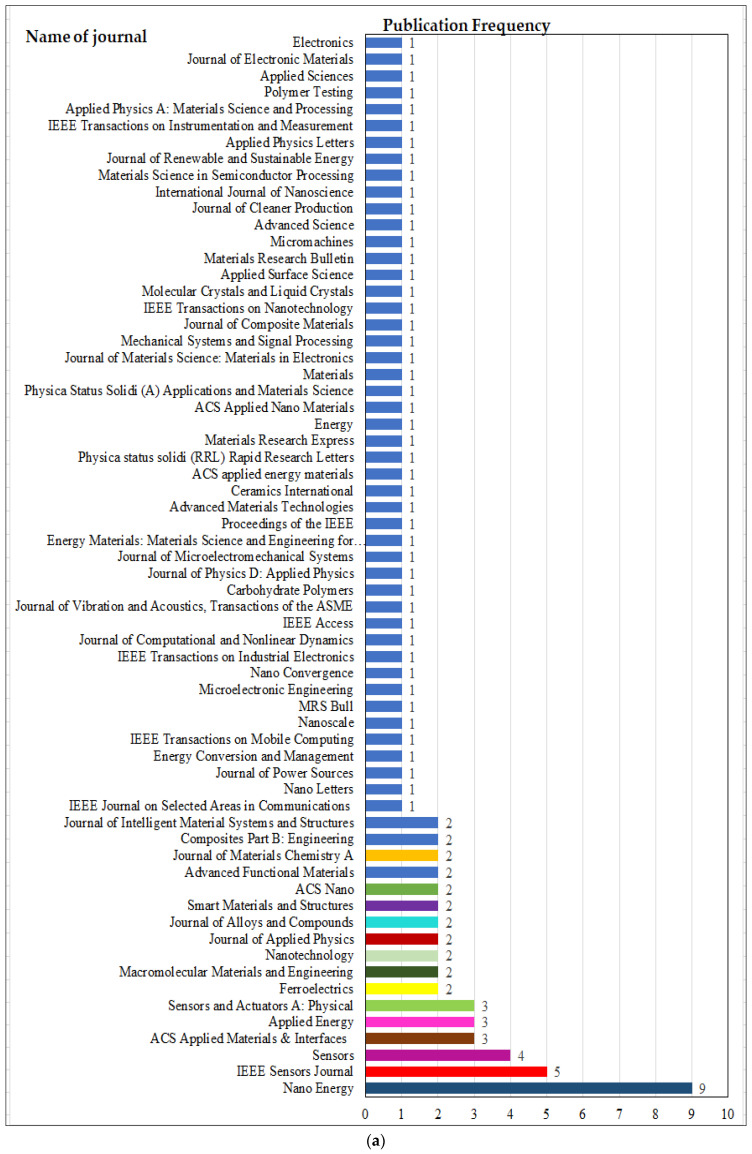

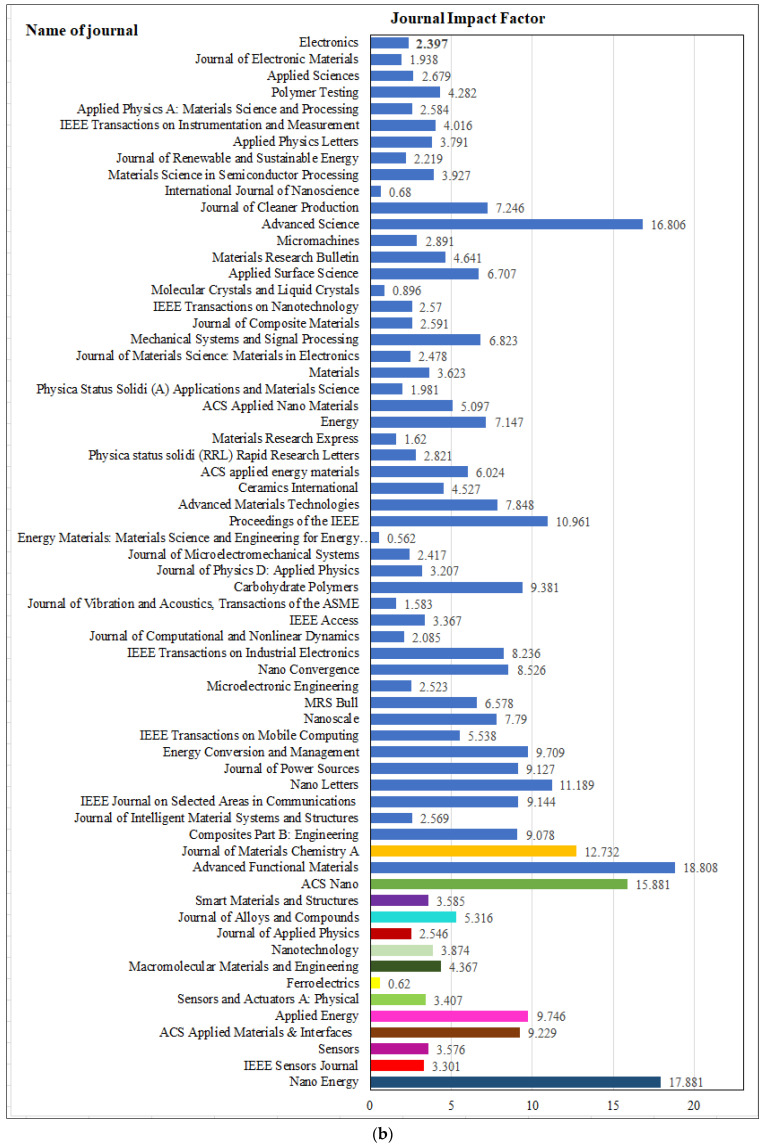

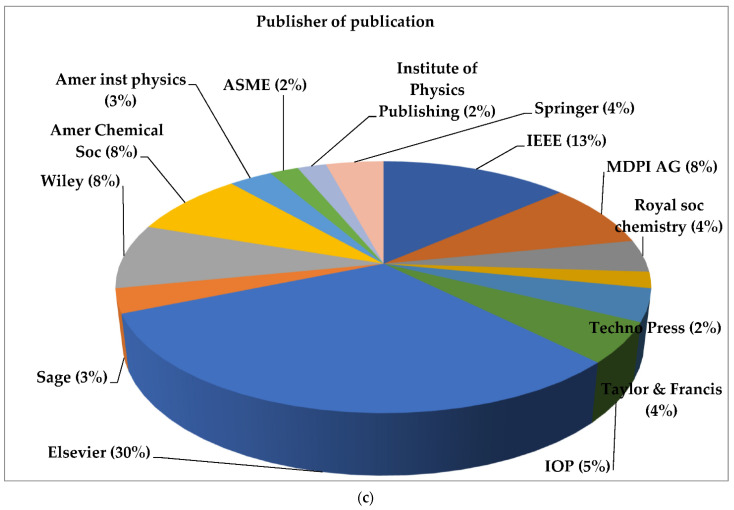
Articles for (**a**) journal analysis, (**b**) impact factor, and (**c**) publishers.

The low-cost PMEH area is connected to a variety of interdisciplinary domains and manuscripts that are published in a variety of journals. The number of publications in specific journals was used to assess the journals that publish PMEH. The Journal of Nano Energy, IEEE Sensors Journal, and Sensors published articles on low-cost PMEH, as shown in Figure 7. This also indicated that Nano Energy (nine papers), IEEE Sensors Journal (five papers), and Sensors (four papers) had the highest number of papers.

Table 3 was created with the study contributions according to the types of documents. As illustrated in Figure 7a, this group solely considers the contributions of the manuscripts that have a frequency of around 51 (51%) of the total 100 documents. Figure 7a displays the lists of journal contributions that are received in the Scopus database for each year (2010–2021). According to Figure 7a, Nano Energy has the most documents (nine publications), IEEE Sensors Journal has five, and Sensors has four publications.

The ACS Applied Materials & Interfaces, Applied Energy, Sensors and Actuators A: Physical, ACS Nano, Journal of Alloys and Compounds, and Nanotechnology also have a publication on low-cost PMEH, applications, etc. Table 3 and Figure 7b show the impact factors that determine the quality of each journal. Figure 7c then looks at the journal’s review according to the publisher. As indicated in Figure 7c, Elsevier has the largest percentage of published manuscripts (30%), followed by IEEE (13%), MDPI AG (8%), the American Chemical Society, and Wiley (8%). On the other hand, IOP has 5%, and Springer, the Royal Society of Chemistry, and Taylor and Francis have 4% of publications.

## 3.4. Analysis of Main Author Contributions

**Figure 8:**
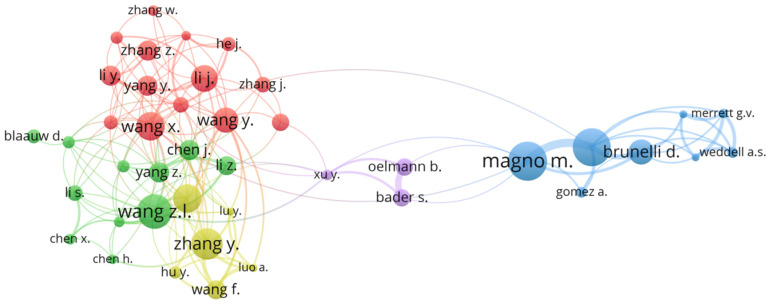
Network mapping of authors with a high frequency of manuscripts.

The most prolific writers were identified by an examination of the scholars who wrote the 24 publications on low-cost PMEH, as well as their co-author networks’ relationships. The minimal number of manuscripts published by an author for this analysis is five; this criterion was met by 35 authors in total. Bernulli d. is the most productive of the 35 authors, contributing eleven low-cost PMEH publications, followed by Magno m., who produced ten. As illustrated in Figure 8, the research group that contributed to the PMEH can be divided into ten groups. Each group’s name was replaced with the leading author, such as Zhang, Wang, and Wang, with each group being highlighted with yellow, green, and red in Figure 8.

### 3.5. Authors Affiliation in PMEH Research

Table 5 shows the top ten affiliations for PMEH studies, which include the Laboratoire de G & eacute, nie Electrique et Ferro & eacute, and lectricit & eacute (six papers); Jeju National University (five papers); Università degli Studi di Catania (four papers); University of Florida (four papers); Institut National des Sciences Appliquées de Lyon (four papers); Georgia Institute of Technology (four papers); Jadavpur University (four papers); University of Michigan, Ann Arbor (four papers); Virginia Polytechnic Institute and State University (four papers); and Université Chouaib Doukkali (four papers).

### 3.6. Top Cited Manuscripts on PMEH

Table 6 shows the top ten most-cited publications in low-cost PMEH over the last 5 years, as measured by the total citations (TC) and average annual citation (ACY) parameters. It is clear from Table 6 that Mao et al. have the highest citations in the last 5 years with a publication titled, “Dynamic Computation Offloading for Mobile-Edge Computing with Energy Harvesting Devices”. On the other hand, Chang, with “Direct-write piezoelectric polymeric Nano generator with high energy conversion efficiency” has the second highest number of citations; Hu et al., with “Recent progress in piezoelectric Nano-generators as a sustainable power source in self-powered systems and active sensors” has the third highest citations. In Table 6, the research gap of these entire publications/research fields is also highlighted.

Based on the selected top-cited publications, Table 7 shows the published manuscripts according to the research type. In the survey, there are five different categories of study. The most common categories of research in published papers include mathematical modelling, algorithm development, data collection, and simulation for EH (35.38 percent). This method is essential for implementing a high-performing system. The second highest type of study (29.61%) is based on EH through piezoelectric material that are synthesized for low-cost applications. A total of 23.1% of articles focus on optimization techniques for sizing, low-cost control, low-cost devices, and low-cost electronics, which provides depth, knowledge, and the main idea of controllable EH studies, while 6.54% of manuscripts are based on detailed review (surveys, critical, state-of-the-art strategic–technical). Currently, researchers have focused more on operational theory, design requirements, and experimental discoveries that demonstrate unique research for low-cost PMEH.

Table 8 shows a more in-depth understanding of the subject as it relates to low-cost PMEH research findings. The subject areas in which the majority of papers were mentioned are listed in this table. The most frequent publication is (30.56%) on the EH system. At 21.53 percent, manuscripts on piezoelectric EH come in second, followed by EH for manuscripts on low-power devices at 13.89 percent. A very limited number of papers (less than 2%) were found in the field of EH for low-cost control systems.

#### 3.6.1. Energy-Harvesting System

Recent advances in sensing technologies, low-power processing, and communication have enabled the IoT, which is expected to become the semiconductor industry’s largest electronics market [44]. A promising idea is to have billions of wirelessly connected sensor devices that can collect and analyze data for a wide range of applications, including fitness and sports, machinery, and health monitoring [34]. In IoT technology, the main trend is to reduce form factors and power consumption while boosting functionality [63]. A fast-growing class of such devices is wearable, where sensors nodes are tightly coupled with the human body [47]. Low-power consumption is critical in wearable devices due to battery weight and size limits, which significantly limit the amount of energy that can be stored. Although integrated circuits have increased their energy efficiency significantly, battery technology has not kept pace in terms of volumetric energy density gains. Furthermore, customer expectations for wearable devices suggest that they will last months, if not years, rather than the daily recharges that are usual in today’s wearable devices [66]. As a result, a low-power design is not enough to make these gadgets genuinely wearable. EH is a new but well-established technology for extending the battery life of wearable devices and allowing continuous recharging of the energy storage while in use [48]. Wearable devices, on the other hand, are constrained in terms of size and weight, and must also be compatible with the human body.

#### 3.6.2. Piezoelectric Energy Harvesting

Piezoelectric nanogenerators that can harvest energy from mechanical energy in the environment are appealing for a variety of applications [37,55,60]. In this research, we show how to transform low-frequency mechanical energy into electric power utilizing piezoelectric ZnO nanorods that are manufactured on a standard paper substrate in a simple and low-cost way [50,82]. This energy-conversion device has extremely high flexibility and piezoelectric sensitivity and can generate output voltages of up to 10 mV and currents of up to 10 nA. It is proved that by regulating the straining rate, the device’s electric output behavior may be switched between four different modes. Furthermore, it is demonstrated that by increasing the device’s size, the electric output may be increased. This EH technique offers a simple and cost-effective platform for capturing low-frequency mechanical energy, such as that which is generated by body movements, for practical applications [68,86].

#### 3.6.3. Energy Harvesting for Low-Cost Applications

Due to increased electricity demand, EH from underused natural-waste energy sources is becoming more widespread. Depending on the environmental conditions, the sources can produce micro- to milliwatts of electricity. Many researchers have focused on micro-level EH in order to deliver power to micro-devices in remote locations [35,136]. The concept results in a significant cost decrease. Once the structure is in place it can generate electricity at a low cost and with little effort, similar to renewable energy sources [72,74,84]. This study examines the mechanical and electronic approaches that are pioneered by different researchers in the PMEH system. Following a thorough examination, it was shown that existing technologies are more or less capable of EH when using piezoelectric elements; nevertheless, the systems’ consistency and stability are not yet up to par. The implementation of the optimization technique to improve the performance of vibration-based PMEH has been considered in this study. Several problems and solutions for next-generation EH using vibration-based piezoelectric devices were the subjects of this review.

#### 3.6.4. Energy Harvesting for Low-Cost Acquisition

The low-cost EH device is based on a rectenna to address the issue in places where battery constraints exist [62,68]. For the EH and IoT applications an energy harvester is built, optimized, constructed, and described, regarding the instability of AC in stable DC power. Human-to-human and human-to-machine communications were recently used by the IoT [34,44]. The IoT allows for device-to-device communication without the need for human interaction, which presents several issues [47]. Due to restricted energy capacities, the machine’s self-sustainability is a major difficulty in this paradigm.

#### 3.6.5. Energy Harvesting for Low-Cost Sensors

A wide range of items can be controlled precisely, locally, remotely, or autonomously with the help of modern low-power sensors [34,37]. Vehicles, appliances, HVAC systems, hospital intensive care units, oil refineries, and military and security systems are all using them. They are essential components for a wide range of global applications. EH technology, for example, permits small, freestanding sensors to operate continuously for long periods of time (even decades) without the need for power lines or battery replacements. This technology significantly improves the problem-solving capabilities of low-power sensors, the cost is low, and its adoption is fast increasing [68,77].

#### 3.6.6. Energy Harvesting for Low-Cost Electronics

Electronic gadgets and systems with wireless capabilities are becoming increasingly popular since they do not require a connection to the main power grid [32,36]. As a result, many gadgets in both the industrial and home environments are completely powered by batteries with the connection lines serving primarily to recharge the batteries. Battery makers have a substantially extended battery life, but they still need to be maintained on a regular basis, and the cost of batteries is increased by thousands of working hours. Furthermore, depleted batteries become garbage that must be recycled. On the other hand, low-cost PMEH is the best energy source for microelectronic devices with the advantages of low cost, ease of maintenance, and little environmental effect [77,85].

#### 3.6.7. Energy Harvesting for Low-Cost Control

EH is significant because it provides an alternative power source for electronic devices in areas where conventional energy sources are unavailable. It has the same advantage in remote sites, underwater, and other difficult-to-reach situations where conventional batteries and energy are insufficient [54,64]. An EH system is controlled by providing an energy storage device with two energy harvesters. The EH is connected to a storage device to transfer the generated power. The transfer characteristics of the EH depend upon the load characteristics.

#### 3.6.8. Energy Harvesting for Low-Power Devices

The amount of power that is utilized by every component in the system must be carefully identified and minimized for independent sensor designs that cannot connect to the AC power main, especially those that must function for lengthy periods of time between battery changes or cannot be accessible for maintenance. It is fantastic if the low-power devices can be powered by a low-cost EH system [34,54,57].

### 3.7. Keywords Analysis

**Figure 9:**
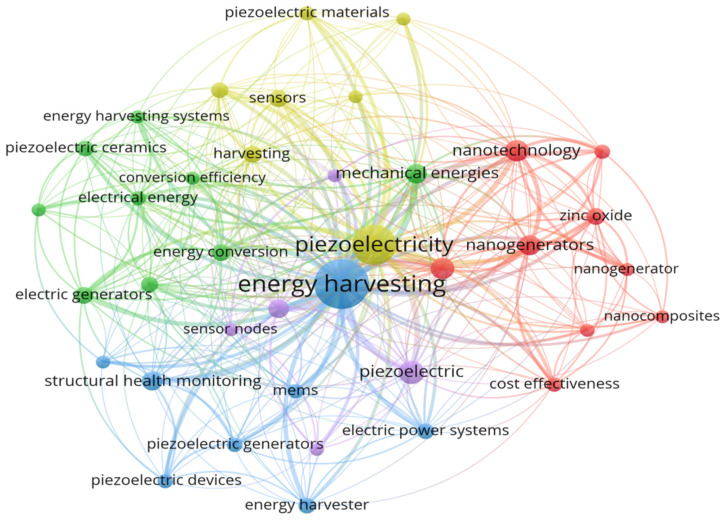
Visualization for the keywords.

The keywords are necessary to identify the paper’s principal focus and make it easier for readers to map [137,138]. The VOSviewer software is used to examine and evaluate the sample keywords in order to highlight the high frequency of keywords and their correlations. The minimum number of keywords was set at 5, and Figure 9 shows 25 of them.

The terms “EH”, “electrostatics”, and “damping” were used the most. Furthermore, “piezoelectricity, piezoelectric devices, natural frequencies, and sensor nodes” were frequently mentioned in the low-cost PMEH literature. The data also show that some significant topics, such as materials and actuator ideas (i.e., piezoelectric, electrostatic, and electromagnetic), as well as methodology, are gaining a lot of attention across several dimensions (i.e., finite element analysis, nonlinear). The interrelationships between those words have also helped to understand low-cost PMEH topics over the last 11 years.

The piezoelectricity circle, for example, is light green in color. We can observe that the highlighted keywords occurred around 2014 based on the color references in the bottom right corner of the Figure 9. In general, the researcher seemed to be more interested in these issues at that time. Furthermore, EH and nanogenerators are two terms that may occur in the future in relation to low-cost PMEH.

**Figure 10:**
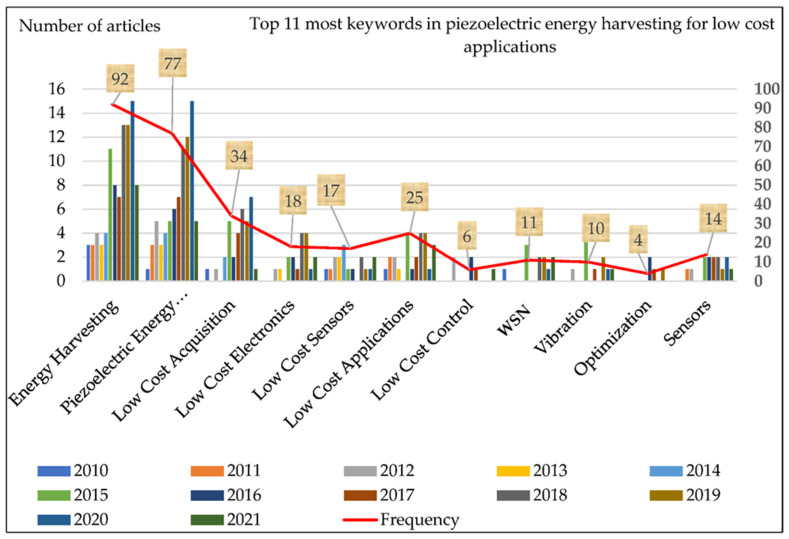
Common keywords analysis topics for 100 papers.

Table 9 shows the top 13 keywords from the chosen database that were used in multiple articles from 2010 to 2021. By examining the highest keywords, the current literature gaps may be detected, and insight into the recent study field can be gained. The three most common phrases in Table 9 are “EH”, “piezoelectric EH”, and “low cost acquisition”. From 2010 to 2021, the most common terms were “low cost electronics”, “low cost sensors”, “low cost applications”, and “low cost control”, demonstrating the increased interest in low-cost PMEH applications. Figure 10 shows the total keyword allocation, as well as a graphical representation of Table 9. In Figure 10, the top 11 most popular keywords over the last eleven years can be seen. The keyword EH is found to be the most frequently utilized. Piezoelectric EH, low-cost acquisition, low-cost electronics, low-cost sensors, low-cost applications, WSN, vibration, optimization, and sensors are among the other important keywords. Table 3 also shows all of the essential terms that are used throughout the manuscript.

## 4. Low-Cost Piezoelectric Energy Harvesting System: Issues and Challenges

The design and deployment of low-cost PMEH is a tough process because there are multiple factors that must be considered, such as economic viability, reliability, power and frequency control, battery-characteristics uncertainty (energy storage system), and environmental considerations [139,140]. However, such challenges can be overcome with an appropriate combination of technological development and implementation. The following is a list of some of the most popular key concerns and challenges in the field of low-power PMEH [141,142]. The scavenging of energy from abundant energy sources faces several challenges, including the availability of the wasted abundant resources in our surroundings, the latest technology of EH, and very good knowledge of low-power electronic devices and the availability of efficient energy-storage systems.

### 4.1. Technical Problems

One of the important parameters in the design of PMEH is the geometry of the piezoelectric cantilever beam [143,144]. Different shapes of the PMEH have different output power levels. The output of the harvesting structure is very low i.e., in the range of µ Watts, full-bridge rectifications are used to obtain the output at usable levels. Another problem is the uncertainty of the vibrations; controllers are used to fix this problem [145,146,147]. Different types of controllers are used with converters. The output also has harmonics which affects the quality of the output power and needs proper filtration. There are different options to store the generated output power; in the market, different batteries and supercapacitors are available and used according to the application and scenario.

### 4.2. Establishment of Electrical Parameters Model

For the design of a piezoelectric EH it is very important to set the standards for (a) the modeling and naming method, (b) terms and formulas, (c) piezoelectric material characteristics, and (d) electrical performance methods. For the modeling and simulation, different techniques and analyzing software are available, but the proper practice is required for the simulations (especially when dealing with COMSOL and ANYSIS, etc.). [144,148]. COMSOL Multiphysics is a simulation and finite element analysis software that runs on any platform. It supports both traditional physics-based user interfaces and partial-differential equations systems. Most of the time, ANSYS is used for volumetric simulations. The same simulations can be carried out with COMSOL, but with different techniques [149,150].

### 4.3. Economic Impact

The most frequently encountered challenge in developing and implementing an effective low-cost PMEH is cost reduction [151,152]. The third most prevalent area of study, according to Table 9 is “low cost acquisition” research. In the last 11 years, about 40% of the articles that were chosen focused on low-cost electronics, low-cost sensors, low-cost applications, low-cost control, and low-power devices. The existing problem could be solved by a cost-optimized system that integrates PMEH with abundant energy resources [153]. With the development of low-cost PMEH the usage of traditional batteries is reducing with time [154]. The cost of batteries as compared to the PMEH is very high, hence the low-cost PMEH is making a positive impact on the electronic industry.

### 4.4. Power Quality Impact

PMEH can be utilized as a backup power source in existing low-voltage electronic devices or as a power source for WSNs etc., [155,156,157]. However, unprecedented fluctuations in power dissipation, voltage, frequency controls, and power management challenges may arise as a result of abundant energy resources integration with low-cost PMEH. For power management, different types of boosters, filters, and controllers are available, but with the application of these circuits the price is increased at the cost of the output power [158].

### 4.5. Environmental Impact

Low-cost PMEHs play an important role in the development of microelectronic devices and reduce the greenhouse effect, compared to fossil fuels. The PMEH have no negative environmental impact except for the energy storage systems, e.g., batteries have a negative impact on the surroundings due to their toxic materials. When batteries fail, the chemicals leak into the earth, contaminating groundwater and surface water [159]. When our ecosystems are contaminated with battery chemicals, thousands of aquatic plants and animals are harmed. PMEH with SC benefits the environment not just by reducing the number of discarded primary, but also by reducing the number of raw materials that are mined [160,161,162].

### 4.6. Storing Energy

Ambient energy sources now play a critical role in integrating the EH system in order to achieve the low-cost PMEH [163,164]. The integration of abundant energy with the current EH industry is gaining popularity, particularly in America and Europe, and Asia. Nonetheless, batteries are still the most common source of electricity in the world. Now, these sources are not usually advised due to their unreliability and greenhouse effect on the environment. The development of low-cost PMEH with abundant energy sources could be the future of EH microelectronic devices [165]. Researchers are currently working on improving the efficiency and output power of PMEH.

## 5. Conclusions and Suggestions

The primary purpose of this research was to summarize low-cost PMEH applications in microelectronic devices, low-cost applications, and low-cost control to advance the existing literature by identifying key knowledge gaps that may be addressed in future research. We identify the most relevant subjects in existing research and uncover emerging and prospective research routes using a bibliometric analysis and qualitative reviews of chosen articles. A total of 100 publications were picked for the analysis and considered for the final review. The findings of the review can be relevant to scholars who are researching the low-cost PMEH.

Despite the increasing literature on PMEH, there are still a lack of publications that provide a comprehensive view of PMEH future advancements, broadening the subject’s understanding and bridging the knowledge gap. As a result, our analysis focused on identifying numerous subjects and research emphases at the intersection of PMEH and low-cost applications. This analysis provides several useful insights. For improved harvesting, the cost and quality of the output power is important. The efficiency of the PMEH system can be increased by the following suggestions:

- By the application of a hybrid method, the output efficiency can be increased. Hybrid EH devices have been proposed in recent years to overcome the energy insufficiency issue of a single energy harvester. A proper hybridization of multiple energy conversion methods not only enhances space utilization efficiency, but also greatly increases the power output;
- The fuzzy controller is capable of correcting fluctuations that are induced by external factors, such as room temperature and convection of voltage;
- Neural networks can have a huge number of free parameters (the weights and biases across interconnected units), which allow them to fit exceedingly complicated data that other models are unable to fit (when trained correctly);
- Harmonics is also a problem that needs to be tackled. Depending on the configuration and application, passive harmonic filters use inductors and capacitors to block or shunt harmonics, causing them to ground. The impedance of an inductor increases as the frequency increases, whereas the impedance of a capacitor decreases;
- In most converter circuits there is a significant amount of switching and power loss in the passive components. Various wide-bandgap (WBG) material compositions, such as silicon carbide (SiC) and gallium nitride (GaN) are currently used in the development of converters because of their capacity to handle high voltages and currents while dissipating low heat. Despite this, the material is unreliable and costly. As a result, future research should place a higher priority on the adoption of these complex materials for PMEH applications;
- In addition to wide-bandgap (WBG) materials, such as SiC and GaN, much attention is now being paid to ultra-wide bandgap (UWBG) materials, such as Al(Ga)N and Ga2O3, since they have a greater power density and can be used in high-power applications. Although UWBG materials are still in the early stages of development, they have the potential to be used as switches in DC-DC converters, which could have several benefits for PMEH applications. As a result, more research should be carried out to determine the best material composition for building a suitable converter for EV applications that is reliable, affordable in cost, and has a high switching frequency;
- The various converter topologies confront high harmonics in output current, low current, voltage stress, and low impedance. Further research is needed to improve the electrical design features, in order to attain high frequency and low converter loss. In addition, investigations on mechanical design optimization should be carried out, in order to improve reliability and accuracy;
- External design schemes are as significant as internal design features when it comes to converters. Improving internal electric-design elements is insufficient to provide the desired results and improvements. Changing the internal electric design, on the other hand, often adds unnecessary complexity and is exceedingly time-consuming and costly. In this regard, numerous investigations are being undertaken in order to produce various passive and active power filters that can improve the performance of converter from the outside. The main benefit of these power filters is that they are simple to construct using low-cost power electronic components and may significantly reduce high harmonics, disturbances, and noises in the converter output signals;
- Due to numerous factors, such as low current stress, easy control mechanism, and high-performance efficiency, the use of multilayer multi-phase bidirectional converters in PMEH technology has increased significantly. However, more research is needed to determine the needs for additional components and complicated analyses in both steady-state and transient settings. Furthermore, the converters have a high-duty cycle sensitivity to varying loading conditions. As a result, it is recommended that efforts be focused on building an integrated design framework in order to improve scalability and reliability;
- Machine learning approaches are now being investigated extensively for predicting and analyzing various forms of power converter problems, such as short circuit and open circuit faults. The ability to accurately detect these defects is important because it can prevent the converters from physical damage and other potentially dangerous situations. As a result, machine learning techniques are becoming increasingly important in the implementation of power converters in PMEH applications.

Furthermore, the results of this review will motivate researchers to continue working on building intelligent converters in the PMEH field with improved design configuration and operational capabilities. The intelligent converters deal with progress in speed, efficiency, and power processing. Overall, this study aids in the development of a strategy for future sustainable PMEH expansion. Our research demonstrates the importance of these publications and their impact in the advancement of low-cost PMEH over time. In terms of global effect, different countries were mentioned. The current study uncovers several novel insights on the role of PMEH for low-cost applications and increasing the efficiency of microelectronic devices.

Our research demonstrates the importance of these publications and their impact in the advancement of low-cost PMEH throughout time. In terms of global effect, different countries were mentioned. The current study uncovers several novel insights on the role of PMEH for low-cost applications and increasing the efficiency of microelectronic devices.

Citation analysis on a certain subject within a specific publication has become a common way to assess a journal’s, author’s, or article’s quality. A systematic review is another way to assess an article’s worth by looking at the author’s affiliation, citations, keywords, and most prestigious journals. The top papers of the 100 most-cited papers are included in the suggested article to provide insight into the historical and present approach of researchers in scientific analysis, and issues related to low-cost PMEH. There are numerous advantages to determining the characteristics of the most-cited papers, including:

- The characteristics of highly cited works in the field of low-cost PMEH for low-cost and microelectronic devices applications can provide future researchers with a clear picture;
- A bibliographical analysis can give researchers a fantastic perspective on a thriving and developing field of research, motivating various devoted researchers to employ current and new technologies to advance a specific research field;
- Researchers and journal editors can use the topmost cited article-analysis to help them review submitted articles.

However, it is expected that identifying and assessing articles that had a significant influence between 2010 and 2021 will help to inform future EH, in order to achieve an independent and efficient power source.

## Figures and Tables

**Table 1:** Summarized analytical evaluation of review manuscripts.

References	Year	Focused Topics	Research Gaps
[22]	2021	A bibliometric analysis on Pediatric Surgery is presented where articles from Web of Science within the years 1986 to 2012 are considered.	Keyword analysis and recent articles were not included in the top 100 most-cited articles.
[12]	2021	Top 100 most-cited articles on dentistry were presented.	The keyword analysis, as well as the most popular terms utilized in various years, recent important papers, and study types were not addressed.
[14]	2020	A bibliometric analysis is presented in the field of medical imaging where Scopus and Web of Science database were used to extract articles.	Because the average citation per year was not taken into account, no recent articles were considered for the analysis.
[23]	2020	100 most-cited articles in the field of general thoracic surgery are extracted from Web of Science database.	A detailed surveying approach is described, but there is no list of keywords from various years or keyword analysis.
[24]	2021	A detail bibliometric analysis of battery thermal management systems is presented, including a detail keyword analysis, surveying methodology and discussion.	The selected list of papers with the most citations was missing. Furthermore, research gaps in the field of studies, as well as contemporary trends are not taken into account.
[25]	2021	A bibliometric analysis on battery storage systems with renewable energy integration is presented and the articles were extracted from Scopus database.	The research gaps, concerns, and challenges of the subject of study are not explored, but a detailed keyword analysis, surveying technique, and recent research trends are mentioned.

**Table 2:** Potential of articles in the Scopus database.

Stages	Filter	Keyword Codes	Number of Manuscripts
1st stage	Energy harvesting system for low-power applications	TITLE-ABS-KEY (energy AND harvesting AND system AND for AND low AND power AND applications)	2549
2nd stage	Low-cost energy harvesting system for low-power applications	TITLE-ABS-KEY (low AND cost AND energy AND harvesting AND system AND for AND low AND power AND applications)	551
3rd stage	Piezoelectric energy harvesting system for low-cost applications	(Piezoelectric AND energy AND harvesting AND for AND low AND cost AND applications)	201
4th stage	Year range (2010–2021)	TITLE-ABS-KEY (piezoelectric AND energy AND harvesting AND for AND low AND cost AND applications) AND (LIMIT-TO (PUBYEAR, 2021) OR LIMIT-TO (PUBYEAR, 2020) OR LIMIT-TO (PUBYEAR, 2019) OR LIMIT-TO (PUBYEAR, 2018) OR LIMIT-TO (PUBYEAR, 2017) OR LIMIT-TO (PUBYEAR, 2016) OR LIMIT-TO (PUBYEAR, 2015) OR LIMIT-TO (PUBYEAR, 2014) OR LIMIT-TO (PUBYEAR, 2013) OR LIMIT-TO (PUBYEAR, 2012) OR LIMIT-TO (PUBYEAR, 2011) OR LIMIT-TO (PUBYEAR, 2010)) AND (LIMIT-TO (EXACTKEYWORD, “Energy Harvesting”) OR LIMIT-TO (EXACTKEYWORD, “Piezoelectricity”) OR LIMIT-TO (EXACTKEYWORD, “Piezoelectric”) OR LIMIT-TO (EXACTKEYWORD, “Costs”) OR LIMIT-TO (EXACTKEYWORD, “Piezoelectric Energy Harvesters”) OR LIMIT-TO (EXACTKEYWORD, “Piezoelectric Energy Harvesting”) OR LIMIT-TO (EXACTKEYWORD, “Sensors”) OR LIMIT-TO (EXACTKEYWORD, “Low Costs”) OR LIMIT-TO (EXACTKEYWORD, “Low Power Electronics”))	189

**Table 4:** Top 10 authors with the highest number of manuscripts in this area.

Rank	Author Name	Current Institution	Country	No. of Articles	No. of Citations	h-Index
1	Magno, M.	ETH Zürich	Switzerland	8	3152	33
2	Tentzeris, M.M.	Georgia Institute of Technology	USA	7	16,749	62
3	Wang, Z.L.	Georgia Institute of Technology	USA	7	239,581	240
4	Pelrine, R.	SRI International	USA	5	10,184	36
5	Benini, L.	Alma Mater Studiorum Università di Bologna	Italy	4	31,881	81
6	Eckerle, J.	SRI International	USA	4	1825	14
7	Georgiadis, A.	Heriot-Watt University	UK	4	5384	34
8	Kim, S.	Pusan National University	South Korea	4	1620	18
9	Kornbluh	SRI International	USA	4	10,023	35
10	Prahlad, H.	SRI International	USA	4	1327	19

**Table 5:** Top 10 affiliations in PMEH for low-power applications research (2010–2021).

No	Author Institutions	Frequency of Articles
1	Laboratoire de G & eacute; nie Electrique et Ferro & eacute; lectricit & eacute	6
2	Jeju National University	5
3	Università degli Studi di Catania	4
4	University of Florida	4
5	Institut National des Sciences Appliquées de Lyon	4
6	Georgia Institute of Technology	4
7	Jadavpur University	4
8	University of Michigan, Ann Arbor	4
9	Virginia Polytechnic Institute and State University	4
10	Université Chouaib Doukkali	4

**Table 6:** Top ten articles based on “highest citation in the last 5 years from 2017 to 2021.

Rank	Author Name	Article Title	Last 5 Years’ Citation	Total Citation Rank	ACY	Advantage	Contribution	Research Gap
1	Chang, C., Tran, V.H., Wang, J., Fuh, Y.K., Lin, L.	Direct-write piezoelectric polymeric nanogenerator with high energy conversion efficiency	512	2	103	PVDF nanofibers have good piezoelectric effect as compared to polyethylene oxides.	Under mechanical stretching the output is repeatable and constant with good efficiency.	Evaporation is a big problem.
2	Mao, Y., Zhang, J., Letaief, K.B.	Dynamic Computation Offloading for Mobile-Edge Computing with Energy Harvesting Devices	839	1	168	Proposed algorithm shows remarkable results.	For offloading, Lyapunov optimization-based dynamic computation algorithm is introduced.	The central processing unit-cycle frequency is an important parameter to be controlled.
3	Jang, S.; Jo, H.; Cho, S.; Mechitov, K.; Rice, J.A.; Sim, S.H.; Jung, H.J.; Yun, C.B.; Spencer, B.F.; Agha, G.	Structural health monitoring of a cable-stayed bridge using smart sensor technology: Deployment and evaluation	166	5	33	Wireless smart sensors help to monitor the civil structures for long periods.	Efficient data management and low cost of monitoring.	70 sensors and 2 base stations are used; the number should be reduced.
4	Dong, Z.; Kennedy, S.J.; Wu, Y.	Electrospinning materials for energy-related applications and devices	151	7	30	Introduction of electro spinning in EH.	The utilization of electro-spinning to generate materials for four main energy-related applications is highlighted in this paper: (1) fuel cells, (2) dye-sensitized solar cells, (3) Li-ion batteries, and (4) supercapacitors.	Attention is still required in the case of new materials.
5	Fan, X.; Chen, J.; Yang, J.; Bai, P.; Li, Z.; Wang, Z.L.	Ultrathin, rollable, paper-based triboelectric nanogenerator for acoustic energy harvesting and self-powered sound recording	219	4	44	A self-powered microphone for sound recording with rolled structure is exhibited for all-sound recording without angular dependence, with the advantages of a large working bandwidth, thin structure, and flexibility.	The triboelectric nanogenerator may be installed on a commercial mobile phone to collect acoustic energy from human speech and use the generated power to charge a capacitor at a rate of 0.144 V/s.	Noise reduction is a problem.
6	Hu, Y.; Wang, Z.L.	Recent progress in piezoelectric nanogenerators as a sustainable power source in self-powered systems and active sensors	200	3	40	The nanogenerator can be utilized as a sustainable power source for self-powered systems and as active sensors, which are two major applications of this technology. Several demos are discussed in this article.	Using ZnO nanowires and a new sandwich structure design, a high-performance piezoelectric nanogenerator may be made in a very simple fabrication procedure with good mechanical stability.	High cost
7	Martinez, B.; Montón, M.; Vilajosana, I.; Prades, J.D.	The Power of Models: Modeling Power Consumption for IoT Devices	161	6	32	This research gives a thorough model for wireless sensor-node power consumption.	This paper introduces a novel paradigm for investigating and assessing energy life cycles in applications. It may be used to predict the precise weight of application parameters in advance, as well as to comprehend the system’s tolerance margins and tradeoffs.	Only deals with parameters that could be empirically quantified.
8	Lee, S.; Bae, S.H.; Lin, L.; Yang, Y.; Park, C.; Kim, S.W.; Cha, S.N.; Kim, H.; Park, Y.J.; Wang, Z.L.	Super-flexible nanogenerator for energy harvesting from gentle wind and as an active deformation sensor	115	10	23	Nanogenerator (NG) with max. output voltage.	This paper describes a super-flexible and conformable NG based on low-cost thin Al-foil electrodes that can not only collect energy from a waving flag but also detect a moving object; when linked to a human face, the skin moves.	Highly flexible material is used.
9	Garain, S.; Jana, S.; Sinha, T.K.; Mandal, D.	Design of in Situ Poled Ce3+-Doped Electrospun PVDF/Graphene Composite Nanofibers for Fabrication of Nanopressure Sensor and Ultrasensitive Acoustic Nanogenerator	138	8	28		Design of efficient ultrasensitive acoustic-nanogenerator.	
10	Thielen, M.; Sigrist, L.; Magno, M.; Hierold, C.; Benini, L.	Human body heat for powering wearable devices: From thermal energy to application	124	9	25	Energy harvester for wearable devices.	This research investigates scavenging human body heat and improving the efficiency of power conversion from the body core to the application.	Requires critical power conditioning.

**Table 7:** Highly cited manuscripts in Scopus database.

Types of Study	Frequency	Range of Years	Citation Range
Mathematical modelling, algorithm creation, data collection and simulation for energy harvesting	92	2010–2021	3–992
Energy harvesting through piezoelectric material synthesized for low-cost applications	77	2010–2021	3–992
Optimization techniques for sizing, low-cost control, low-cost devices, and low-cost electronics	60	2010–2021	3–992
Review (surveys, critical, state-of-the-art strategic–technical)	17	2011–2021	3–352
Development, evaluation, and experimental prototype low-cost sensors	14	2010–2021	3–355

**Table 8:** A total of 50 manuscripts with highest citations in various fields of research.

Subject Area	Rank of the Manuscript According to Table 3	Publication Rate	Citation Range
Energy-harvesting system	[31,32,34,35,36,37,44,45,46,47,48,51,52,55,56,57,58,59,60,61,62,63,64,65,66,67,68,69,70,71,72,73,74,75,76,77,78,79,80,81,82,84,85,86,87]	44	20–992
Piezoelectric energy harvesting	[31,35,37,45,46,49,50,53,55,56,57,59,60,62,65,68,69,70,72,73,75,76,78,79,81,82,83,84,85,86,87]	31	20–992
Energy harvesting for low-cost applications	[34,35,44,47,50,51,61,72,73,74,81,82,83,84]	11	23–352
Energy harvesting for low-cost acquisition	[31,52,53,55,57,58,60,62,66,67,68,69,71,75,76,79,86,87]	18	20–992
Energy harvesting for low-cost sensors	[34,37,45,48,56,58,65,68,70,74,77,78]	10	33–355
Energy harvesting for low-cost electronics	[32,36,44,47,63,77,80,85]	8	20–844
Energy harvesting for low-cost control	[54,64]	2	54–85
Energy harvesting for low-power devices	[32,34,44,47,48,50,51,54,57,59,61,63,64,65,67,71,74,76,78,82]	20	24–844

**Table 9:** Relevant 13 most common keywords used in different articles from the year 2010 to 2021.

Rank	Keywords	2010	2011	2012	2013	2014	2015	2016	2017	2018	2019	2020	2021
1	Energy Harvesting	[31,34,51]	[35,103,128]	[52,64,93,94,110]	[45,70]	[37,56,75,76,78,97]	[36,44,58,61,67,71,77,87,115,122,127]	[7,32,46,59,96,100,109,116]	[47,57,62,79,84,102,108]	[48,55,63,65,66,68,69,86,88,89,92,99,121]	[72,74,80,85,91,95,98,101,107,114,120,132,133]	[3,60,82,83,90,105,111,112,113,119,124,126,129,131]	[1,4,81,104,106,117,118,125]
2	Piezoelectric Energy Harvesting	[31]	[35,103,128]	[50,73,93,94,110]	[45,70]	[37,56,75,76,78,97]	[87,115,122,127]	[7,46,59,96,100,109,116]	[49,57,62,79,84,102,108]	[53,55,65,68,69,86,88,89,92,99,121]	[72,80,85,91,95,98,101,107,114,120,132,133]	[3,60,82,83,90,105,111,112,113,119,124,126,129,131]	[81,104,106,117,125]
3	Low-Cost Acquisition	[31]		[52]		[75,76]	[58,61,67,71,87,122]	[96,109]	[57,62,79,108]	[53,55,66,68,69,86]	[101,107,114,120,133]	[60,90,105,112,119,129]	[106]
4	Low-Cost Electronics						[36,44,77]	[32]	[47]	[63,121]	[80,85,98]	[126,131]	[4]
5	Low-Cost Sensors	[34]	[103]	[93,94]	[45,70]	[37,56,78]		[59]		[48,65]	[132]	[124]	[104,118]
6	Low-Cost Applications	[51]	[35,128]	[50,73]		[97]	[115,127]	[100]	[84,102]	[88,89,99,121]	[72,74,91,95]	[3,82,83,111,119]	[81,117,125]
7	Low-Cost Control			[64,110]				[7,116]	[54]			[1]	
8	WSN	[34]					[36,44,115,122]			[48,66]	[72,74]	[124]	[1,4]
9	Vibration			[64]			[71,77,122]		[84]		[107,114]	[126]	[106]
10	Optimization							[7,116]	[47]		[72]		
11	Sensors		[128]	[73]			[58,87]	[46,100]	[49,54]	[86,89]	[91]	[83,129]	[104]
12	Low-power devices	[34,51]		[50,52,64]	[45,70]	[37,76,78]	[36,44,58,61,67,71,122]	[7,32,46,59,100]	[47,49,54,57]	[48,53,63,65,69,92,99]	[74,85,98,101,120]	[1,3,82,112,113,126,129,131]	[118,125]

## Data Availability

Not applicable.
